# Beyond the Surface: A Consensual Qualitative Research into the Multifaceted Nature of Bullying

**DOI:** 10.3390/bs15121631

**Published:** 2025-11-27

**Authors:** Laura Menabò, Debora Ginocchio, Felicia Roga, Eleonora Renda, Annalisa Guarini

**Affiliations:** 1 Department of Psychology “Renzo Canestrari”, University of Bologna, Viale Carlo Berti Pichat, 5, 40127 Bologna, Italy; felicia.roga@unibo.it (F.R.); eleonora.renda3@unibo.it (E.R.); annalisa.guarini@unibo.it (A.G.); 2 Faculty of Law, University of Modena and Reggio Emilia, Via San Geminiano, 3, 41121 Modena, Italy; debora.ginocchio@unipr.it

**Keywords:** bullying, qualitative research, focus group, definition, roles, coping strategies

## Abstract

Bullying is a significant social issue, yet research often relies on quantitative methods. Our study aimed to gain a deeper insight by giving students a voice in expressing their experiences and perceptions, focusing on how youth define and perceive bullying, the different roles involved, and the coping strategies they identify. We conducted 16 focus groups with 220 Italian students, using the Consensus Qualitative Research method. Seven key domains emerged, with core ideas classified by frequency: general (>75%), typical (25–75%), and variant (≤25%). In “Characteristics of bullying,” power imbalance was general, intentionality was typical, and repetition was variant. In “Bullying behaviors,” physical and verbal bullying were general; relational bullying was variant. Regarding “The bully,” moral disengagement and compensation were general, retaliation was typical, and intimidation was variant. For “The victim,” perceived weakness and well-being were general, ethnic victimization was typical, and victim blaming was variant. In “Other roles,” pro-bullies and passive bystanders were typical; defenders were variant. “Victim’s coping strategies” included nonchalance, distancing, and seeking social support as general; retaliation as typical; and talking with the bully as variant. Finally, “Bystanders’ coping strategies” included protecting the victim (typical) and self-protection (variant). These findings offer a nuanced perspective on bullying and inform more targeted interventions.

## 1. Introduction

Bullying represents a priority for global researchers, policymakers, and educational institutions (Arseneault, 2018; Casper, 2021). Indeed, being involved can seriously harm students’ physical, emotional, and psychological well-being, requiring urgent attention (Casper & Card, 2017; Menabò et al., 2024). However, while there is a significant history of research into bullying, as Smith et al. (2021) noticed, over three-quarters of that research is quantitative, despite calls for more qualitative and mixed-method studies (e.g., Patton et al., 2017). This heavy reliance on quantitative research has somewhat constrained a more nuanced understanding of bullying (Bosacki et al., 2006; Smith et al., 2021).

Regarding the definition of bullying, it is described as a pattern of aggressive behaviours perpetrated by one or more students against a peer who is less capable of defending themselves. This definition encompasses three key criteria: the imbalance of power, the repetition, and a deliberate intent to cause harm (Olweus, 1992; Smith et al., 2013). Among these, power imbalance has increasingly been recognized as the most distinctive feature, as it highlights the social nature of bullying and distinguishes it from general aggression (Andrews et al., 2023; Volk et al., 2014), reflecting group dynamics and the interaction between individual and contextual factors (Horton, 2020). Power may manifest in various forms, physical, psychological, social, or economic, and is often co-constructed and sustained by peer dynamics, such as pro-bullies or passive bystanders’ behavior (Salmivalli, 2010; Kärnä et al., 2010).

Nevertheless, over time, scholars have raised doubts about the adequacy of the key criteria in capturing the complexity of bullying (Menin et al., 2021; Skrzypiec et al., 2023). For instance, Menin et al. (2021) noted that students perceive dominance as the primary factor in defining bullying, followed by reported harm and deliberateness, while repetition is not deemed significant. Another quantitative survey by Skrzypiec et al. (2023) found that only slightly more than half of the participants correctly identified instances of bullying or non-bullying behaviour. Interestingly, all students who felt bullied, regardless of meeting the three criteria, reported harm from the experience. In this regard, qualitative research can assist scholars in shedding light on what students perceive as bullying by offering a thorough exploration and interpretation of individual experiences. Indeed, qualitative studies suggest that the definition of bullying could be more nuanced and the proposed criteria could not be exhaustive or comprehensive since other elements, like the victim’s perception of harm, could be enough to be considered bullying (Celik et al., 2023; Guerin & Hennessy, 2002; Jeffrey & Stuart, 2020). In semi-structured interviews, for example, Guerin and Hennessy (2002) found that the most important criterion was whether the victim was upset by the episode regardless of the bully’s intention. In a qualitative study by Jeffrey and Stuart (2020), students demonstrated an understanding of harm, power dynamics, and intentionality in their definitions of bullying. However, the victims’ perspective emerged as the most significant: their assessment of the situation, reactions, and emotional experience.

Bullying can assume different forms, including physical and verbal, known as overt bullying, as well as relational bullying, which is considered covert due to its subtle and often harder-to-detect nature (Hellström & Lundberg, 2020). However, some research has revealed that while students can differentiate between these types, they often focus primarily on overt forms of bullying (e.g., Bauman et al., 2020). Indeed, a quantitative study (Bauman et al., 2020) found that verbal bullying and relational bullying are considered less serious than physical bullying, likely because these forms are spread and normalized. This tendency was also confirmed by qualitative studies (Hellström et al., 2015) that showed that students, especially from primary and lower secondary school, may have difficulty associating bullying with relational behavior. For instance, Grade 7 students were less likely than Grade 9 students to classify social exclusion, such as “constantly ignoring someone or refusing to talk to them” or “deciding during recess who can (or cannot) join games or other activities”, as bullying (Hellström et al., 2015, p. 3). In this regard, more qualitative research could be crucial in better understanding the nuances of bullying, especially in terms of how relational bullying is perceived and experienced by students.

Qualitative research can also offer valuable insights into the social dynamics of bullying, providing a rich framework for delving into the intricate relational aspects involved. Indeed, while quantitative studies have delineated various roles within bullying relationships—such as pro-bullies aiding the bully, defenders supporting the victim, and passive bystanders observing (Salmivalli et al., 1996)—students’ broader social awareness of bullying is less frequently observed. For instance, a quantitative study by Guarini et al. (2019) found that only about 1% of students aged 11–14 mentioned roles beyond the traditional bully-victim dichotomy when asked who is involved in bullying dynamics, suggesting a limited awareness of the multifaceted social dynamics at play. Qualitative research provides further insight into this aspect. Thornberg and Knutsen (2011) conducted a mixed-method study where students were prompted to provide personal explanations of bullying. The findings indicated a tendency among youth to attribute bullying to individualistic reasons, focusing on the characteristics of the bully and victim rather than considering broader social or systemic factors. Similarly, Ybarra et al. (2019) found in focus group discussions that students frequently described victims as quiet, unpopular, shy, or having a history of negative behavior, without recognizing the role or responsibility of other students or their potential impact on the situation. These findings suggest that students tend to view bullying as an issue limited to the bully and the victim. Overall, the studies highlight the need to deepen our understanding of the various roles within bullying dynamics, emphasizing the importance of collecting more qualitative data to fully capture these complexities.

Another important aspect to consider in bullying dynamics concerns how people respond to these issues, namely coping strategies. Coping strategies have been found to mediate the relationship between victimization and stress symptoms, indicating their importance in managing the impact of stressful experiences (Newman et al., 2011; X. Wang et al., 2024). However, the effectiveness of the different coping strategies is uncertain and results are inconsistent; for instance, seeking social support has been found to increase victimization in some studies (Andreou, 2001; Bijttebier & Vertommen, 1998), while others show the opposite (Davidson & Demaray, 2007; Demaray & Malecki, 2003), or no clear associations. Additionally, avoidant responses, such as emotional and cognitive distancing, may worsen victimization, while deliberate nonchalance has been shown to reduce it (Salmivalli et al., 1996). Given these mixed results, further research is essential to better understand coping strategies and enhance our ability to support students effectively. For example, interviews with chronically bullied students (Tenenbaum et al., 2011) revealed that victims primarily employed problem-focused coping strategies like directly addressing a problem by thinking about it, analyzing possible solutions, and sometimes implementing a solution, albeit with perceived limited success. Other qualitative studies showed that many barriers exist in seeking help, including fear of retaliation from the bully (Celik et al., 2023) and doubts about adults’ ability to understand and intervene effectively (Athanasiades & Deliyanni-Kouimtzis, 2010; Thornberg & Delby, 2019). In addition to victims’ coping strategies, it is also crucial to consider the role of bystanders, whose responses can significantly shape bullying dynamics. Research has shown that bystanders may adopt a variety of defending behaviors, such as directly intervening with the bully, comforting the victim, or reporting the incident to adults (Lambe & Craig, 2020; Z. Wang et al., 2023). When bystanders choose to defend, bullying tends to decrease, as the frequency of such behaviors in the classroom is reduced (Salmivalli et al., 2011). However, the majority of bystanders engage in non-intervention strategies, such as ignoring the situation or pretending not to notice (Jenkins et al., 2022). This passivity may stem from various factors, such as fear of becoming a target, concerns about losing popularity or friendships, doubts about one’s own effectiveness, or a lack of personal connection with the victim (Spadafora et al., 2020), and evidence indicates that when bystanders remain passive, bullying is not mitigated but rather exacerbated (Thornberg et al., 2022).

Despite extensive research, most studies have predominantly relied on quantitative methods, leaving students’ own voices underrepresented in the debate on how bullying is defined, perceived, and managed. Our study addresses this gap by adopting a qualitative approach that foregrounds students’ perspectives, offering insights into how they conceptualize bullying, interpret the roles involved, and describe the strategies used to cope with it. Specifically, the study engages with three key areas of reflection. First, the dominant definition of bullying, traditionally anchored in the criteria of power imbalance, intentionality, and repetition, has been increasingly questioned in recent scholarship, suggesting that these elements may not fully reflect youths’ lived experiences. Second, although research has highlighted the complexity of bullying as a group phenomenon, involving pro-bullies, defenders, and passive bystanders, students often appear to frame it primarily as a dyadic interaction between bully and victim, leaving other roles less visible. Understanding whether these roles are truly absent from students’ conceptualizations, or whether they are simply less salient in their narratives, requires direct qualitative exploration. Third, while the literature has mapped a wide range of coping strategies, from problem-focused to avoidant responses, little is known about which of these strategies are actually recognized, valued, and employed by students in their daily lives. Building on these reflections, the overarching research questions guiding this study were designed to capture youths’ perspectives in these three domains: (1) How do youth define and perceive bullying? (2) How do youth perceive the different roles in bullying, including bully, victim, and bystanders? (3) Which coping strategies do youth identify as meaningful and accessible in their daily lives? By exploring these questions, this study offers a nuanced perspective on youths’ lived experiences, thereby informing more comprehensive and socially grounded interventions.

Given the complex and evolving nature of bullying, along with the recognition that existing research predominantly emphasizes quantitative analyses, which leaves open the debate regarding central features (definition, types of behaviour, roles, coping strategies), our study aims to gain a deeper insight into the phenomenon through a qualitative approach. Specifically, we aim at exploring bullying through focus groups, giving voice to students to express their views in their own words, revealing the complexities of social interactions and the meanings they ascribe to their experiences (Morgan, 1997). By discussing their experiences and perceptions of these phenomena, we hope to uncover new perspectives in the understanding of bullying and the social aspects (e.g., the roles involved) and their views on the effectiveness of various coping mechanisms.

## 2. Materials and Methods

### 2.1. Participants

A total of 16 focus groups were gathered across 7 secondary schools in the Emilia Romagna region (Italy) during 2022. Each focus group corresponded to a class within the first and second year of lower secondary school (grades 6 and 7). The final sample consisted of 220 students, with 114 males (52%) and 106 females (48%). A total of 20 students (9%) were of foreign origin. The mean age of the participants was 11.30 years (*SD* = 0.6).

### 2.2. Procedure

The discussions within the focus groups were facilitated through semi-structured questions. The questions were deliberately simple to elicit diverse and wide perspectives on bullying and to mirror our research questions. These questions included:

1. What is bullying for you?
2. Who do you think is involved in bullying?
3. Do you think there are ways to deal with this phenomenon?

All focus group sessions were conducted by at least one of the authors of this paper. The focus groups were recorded and then verbatim transcribed. To ensure methodological rigor in our qualitative approach, we adhered to the guidelines proposed by Hill et al. (1997) in employing Consensual Qualitative Research (CQR). Our research team consisted of five individuals. Four members (the primary team) were responsible for conducting domain exploration, identifying core ideas, and carrying out the analysis. Additionally, an independent auditor provided an external review of the work. The primary team comprised different experts in the field of bullying: a trainee psychologist, a research psychologist, a psychologist, and a psychotherapist. The independent auditor is a psychologist and a senior expert scholar on bullying. During initial meetings, the group deliberately emphasized the importance of equitable participation, ensuring that all members felt empowered to express their perspectives, as acknowledging the influence of interpersonal power dynamics was imperative (Hill et al., 1997, 2005). In preparation for the research, the first author extensively studied CQR analysis, drawing insights, and devised a coding scheme in Italian to mitigate language barriers among team members not fluent in English. Subsequently, the entire team convened for two sessions to practice and refine their skills using this approach. According to Hill et al. (1997), data analysis consists of three steps. First, the team created the Domains, namely broad topics to cluster interview data. Each primary team member completed this step within each focus group assigned. The second step consisted of creating the core ideas, namely summaries capturing the fundamental essence of the information provided more clearly and concisely to group data within domains. Finally, the cross-analysis was used to develop categories that describe the common themes reflected in the core ideas.

#### 2.2.1. Domains and Consensus Version

The domain list reflects the meaningful topic areas covered in the focus groups. For the creation of the domains, we chose to follow the suggestion of Hill et al. (1997, 2005) and not rely directly on a list of domains decided a priori, but reread all the focus groups to create the domains from scratch. Thus, the initial phase involved individual data segmentation into domains by the primary team members, followed by collaborative efforts to reach a consensus on multiple cases. Specifically, each team member independently segmented and coded three focus groups, with the aim of stimulating comparative reflection. Subsequently, the proposed domains were discussed collectively until consensus was reached, ensuring coherence and reliability. A domain was retained only if it was approved by the team and appeared in at least two focus groups, a criterion that allowed us to distinguish shared content from isolated ideas. Next, the team applied the preliminary list to another transcript and modified it as needed to fit the transcript. We continued this process with new transcripts until no new domains emerged. Once the list was consolidated, the first author completed the coding of the remaining data, which was subsequently reviewed and discussed by the entire team to guarantee consensual agreement. It is important to note that a single statement could potentially convey multiple meanings and therefore be categorized in more than one domain. For instance, the statement “*Bullies are stronger and choose victims who are thinner or have physical difficulties*” was classified within the domains of “*Characteristics of bullying*”, “*The Bully*”, and “*The Victim*”. We then asked the auditor to review the domain list, and we excluded data that were irrelevant to the focus of the study.

#### 2.2.2. Core Ideas

Core ideas “are summaries that capture the essence of the interview’s statements in fewer and often clearer words” (Hill & Knox, 2021, p. 45). In other words, the core ideas process was implemented by paraphrasing the narrative to compare core ideas across different cases. To create the core ideas, it was important to maintain fidelity to the original data, devoid of assumptions or interpretations, while aiming to eliminate redundancies. To do this, primary team members independently crafted the core ideas using the participants’ exact wording and subsequently discussed them to achieve consensus. This stage involves a methodical “editing” process, condensing participants’ expressions into a concise, clear, and comparable format across cases. This means ensuring consistency in pronouns, removing repetitions, and filtering out irrelevant aspects of interview responses, distilling them to the fundamental core of the message. For example, these words from a student, “*…the bully is a person who looks angry on the outside but inside, yes, has a broken and sad heart.*”, have been summarized in the following core idea: “*The bully looks angry on the outside but inside has a broken and sad heart*”.

Once a shared understanding of the core idea process was established and all the members felt they were trained within the initial cases, we followed a rotation strategy: one team member drafted the core ideas while the rest of the team served as internal auditors, reviewing and challenging the core ideas to ensure their accuracy and quality. Finally, the version was returned to the auditor, who provided her feedback.

#### 2.2.3. Cross Analysis

Following the auditor’s feedback, the primary team consolidated all the core ideas from each domain into a single document, moving away from the original manuscript. As recommended (Hill et al., 2005), we maintained the case and speaking turn numbers linked to each core idea, allowing easy reference to the original transcript. Our team collaboratively reviewed all the core ideas within each domain to identify overarching themes and develop a comprehensive category structure. This process of categorizing evolved dynamically; as we sorted the core ideas, our initial list of categories was refined until we achieved a definitive set of categories for each domain. During the concluding phase of our cross-analysis, each category was categorized as General, Typical, or Variant. A category was labeled Variant if it was present in 1–4 focus groups (≤25% of the total focus groups), Typical if it appeared in 5–13 focus groups (26–75%), and General if it was found in 14–17 focus groups (present in more than 75% of the total focus groups).

## 3. Results

Six key domains, with their respective categories, were identified and are now outlined, specifying whether each category is classified as general, typical or variant (Table 1). In response to the first question about what bullying is, two main domains emerged: Characteristics of Bullying and Bullying Behaviors. When asked who is involved in bullying, students provided extensive responses primarily focusing on the bully and the victim, though other roles were mentioned as well. Due to the significant amount of data on the bully and the victim compared to other roles, we decided to create two separate domains for The Bully and The Victim, which also include themes related to the motivations behind these roles. In contrast, passive bystanders, pro-bullies, and defenders were grouped under a third domain, The Other Roles, as there was notably less information about them. Finally, two additional domains emerged from the question on how to handle these situations: Victims’ Coping Strategies and Bystanders’ Coping Strategies.

The original language of the focus group transcripts was Italian; therefore, the student quotes below are English translations made by the first and second authors to facilitate the reader’s understanding.

### 3.1. Domain: Characteristics of Bullying

The first domain is related to the characteristics of bullying. Within this domain, three categories have emerged. The first is general and consists of recognizing the dynamics of dominance and power imbalance, while intentionality in bullying behaviours emerged as a typical category. Finally, a few groups mentioned that bullying incidents continued for an extended period, so this aspect of repetition was categorized as a variant category.

#### 3.1.1. General

**Category: Dominance and power imbalance.** In line with the definition, students perceive bullying as a power inequity between the bully and the victim where the bully exerts control and dominance over the victim. This imbalance could be due to physical strength or a psychological sense of authority. Interestingly, for students, this power imbalance does not have to be objectively real. It may be that the bullies feel stronger, even if they are not, creating a perceived power imbalance that can be either fake or real.

*“Bullying is characterized by a stronger person picking on someone who is weaker and more fragile than he is”*

(Focus group 11)

*“Is the bully pretending to be stronger than the victim or is he really…”*

(Focus Group 5)

*“In bullying, bigger people, even physically, pick on people who are more fragile or smaller than they are”*

(Focus Group 3)

*“The bully picks on the victim because she/he is a weaker person than she/he is, and he knows that he can insult her or raise his hands by having the upper hand.”*

(Focus Group 2)

#### 3.1.2. Typical

**Category: Intentionality.** Some students point out that the bully’s physical, verbal, or psychological behaviors are intended to offend and cause harm or discomfort to the other person. These are deliberate and premeditated actions, and not done “by accident” because the bully realizes the consequences they may have.

*“Bullying is when one person insults, perhaps beats another person to make him/her feel bad.”*

(Focus Group 17)

*“In my opinion (bullying is) physical or psychological acts towards someone for my own power… that is, I want to beat up that guy because I want to […] I really program it.”*

(Focus Group 9)

*“I think bullying is a wrong thing and it can target anybody…and especially if you’re a bully it means you’re not doing it randomly anyway…”*

(Focus Group 1)

#### 3.1.3. Variant

**Category: Repetition over time.** Students refer to bullying incidents that have persisted for an extended period. These are not necessarily particularly serious actions, but they occur daily, making them difficult for the victim to endure.

*“So, in my opinion, bullying is something that happens over and over again, that is, it doesn’t happen once, but it happens continuously”*

(Focus Group 16)

*“Me (they teased) in elementary school, until fifth grade, then let’s say the classmates grew up more…um, they teased me because of my last name”*

(Focus Group 10)

*“In my opinion (bullying) is a repetition of events, […] even small ones, of several people usually toward another one alone, repeated that make one nervous” “What kind of events? Can you give an example?” “Putting your classmate’s things where he can’t reach to get them, doing it every day…I don’t know.*

(Focus Group 8)

### 3.2. Domain: Bullying Behaviours

When questioned about what bullying is, students refer to a multifaceted set of behaviors that may cause inner as well as outer pain. Physical and verbal aggressions emerged as “general” core ideas, while indirect manifestations aimed at social isolation emerged as “variant”.

#### 3.2.1. General

**Category: Physical harm.** Students acknowledge that bullying encompasses various forms of harm, including physical aggression, like hitting or pushing. These behaviors often leave not only outward physical marks but also cause deep inner wounds.

*“Bullying…um…is… yes, in my opinion, is offenses…they hit, they target the smaller, less strong ones, because then at least they can win”*

(Focus Group 5)

*“The bully aims to do either physical or psychological harm, for example, physical, he may kick or shove…”*

(Focus Group 12)

*“Bullying are physically painful things…that hurt you both inside and outside”*

(Focus Group 9)

**Category: Verbal aggression.** Bullies may intimidate the victim with insults and threats, or ridicule him/her in front of others by teasing and using derisive language.

*“I think bullying is when one person mocks, teases another, because he/she thinks [that person] is weaker than him/herself”*

(Focus Group 3)

*“Bullying is when a person, um […] threatens or insults…um…or embarrasses another person in front of everyone”*

(Focus Group 2)

*“Bullying to me is a set of people threatening other people, or several people or a single person eh… offending them…”*

(Focus Group 7)

*“Bullying is when a person insults another person to make him/her feel bad”*

(Focus Group 9)

#### 3.2.2. Variant

**Category: Social exclusion.** Some students refer to more subtle and indirect bullying behavior, namely social exclusion. The victim may not be involved because of certain characteristics that would not make him/her “appropriate” for a group.

*“[Bullying] is when a boy or girl excludes a person from a group, they make fun of him/her”*

(Focus group 11)

*“[…] I was a little fatter than the others, right? So, I was a little slower, so I was excluded and put among those who play less”*

(Focus group 1)

*“[The bully] feels hatred toward another person, makes him/her turn away from his/her friends”*

(Focus group 14)

### 3.3. Domain: The Bully

The third domain involves the role of the bully. Within this domain, we identified two general core ideas—the compensation and moral disengagement perspectives—that are prevalent among students. A typical core idea (the risk-avoidance perspective) and a variant core idea (instilling fear) were also noted. Overall, while the first three core ideas explore the motivations behind bullying, the last highlights the pervasive fear that bullies can instill in both peers and adults.

#### 3.3.1. General

**Category: The compensation perspective.** Focus groups have revealed that a bully’s actions often stem from a sense of insecurity or revenge, which can originate from family issues or a desire to feel appreciated by friends. Interestingly, this motivation aligns with the compensation perspective (Gondolf, 1985), which suggests that aggressive people target weaker individuals to compensate for their own vulnerabilities and boost their self-esteem.

*“I think bullying is a bad thing…but the bully maybe has problems in his family or his own life, so sometimes it’s not really pure nastiness; there are reasons why he does it.”*

(Focus Group 9)

*“…a person or people who have problems of their own….and try to create problems for others for revenge”*

(Focus Group 16)

*“For me, the bullying is the individual who has had a problem outside of school…and he’s pouring it out on that person because he has motives that spill over…”*

(Focus Group 2)

*“Bullying can also be a form of defense, precisely from a family or other situation that is turned on other people”*

(Focus group 7)

In this regard, the bully’s aggressiveness is also interpreted as a means of venting the bully’s frustration or anger. It might not necessarily be directed at the victim personally, but it is used to release emotions.

*“For me bullying is when a group of people or otherwise one person beats or otherwise offends another boy or girl eh […] for fun […] they exercise their anger against others”*

(Focus Group 17)

*“In my opinion, bullying is people, a person […] who have problems of their own […] and they try to create problems for others to let off steam”*

(Focus Group 1)

In addition, some bullies engage in aggressive behavior to attract attention from friends. They might seek to assert dominance or seek recognition or significance among their peers through these actions.

*“Maybe sometimes the bully gets into bullying, I don’t know, to make his friends laugh…maybe, I don’t know […] he gets into teasing the weaker one, so his friends laugh, and he is happy”*

(Focus Group 4)

**Category: Moral disengagement perspective.** Some students justified bullying behavior as a response to their inability to tolerate certain behaviors or characteristics in their victims. They may perceive the victim’s actions or traits as intolerable, which justifies bullying. This idea aligns with the concept of moral disengagement, where some individuals who bully others may be psychologically motivated to view their victims as “mean” or “guilty,” activating moral disengagement mechanisms to justify their actions (Thornberg et al., 2020).

*“Bullying is defined as aggressive behaviour, both physical and psychological, enacted against a person whom we perhaps cannot stand”*

(Focus Group 2)

*“In my opinion, bullying recalls both physical and psychological mistreatment towards maybe a certain person who you don’t like…”*

(Focus Group 7)

*“In my opinion, a little bit all bullying starts from the person who is getting the threats or insults because they probably said something bad…”*

(Focus Group 5)

#### 3.3.2. Typical

**Category: The repercussion perspective.** Bullying is sometimes justified by considering the potential risks and consequences if the bully were to be challenged by someone stronger. Unlike the compensation perspective, and akin to rationalization, this viewpoint suggests that the bully’s behaviour is shaped by a strategic evaluation of risks and a desire to assert dominance and control within their social sphere.

*“Bullying is not wrong because you hurt the other person but because you run the risk of catching someone stronger”*

(Focus Group 17)

*“The bully is actually an insecure person, who behaves this way because he or she is in turn afraid of being targeted by others…”*

(Focus Group 11)

#### 3.3.3. Variant

**Category: The bully scares!** Bullies not only intimidate their peers but also instill fear among adults who may feel uncertain about how to address the situation effectively. This broader impact suggests the perceived bully’s ability to create an intimidating atmosphere beyond just their peers.

*“[…] we run to go to the bus because every time they (the bullies) take the front seats to get off earlier…you can’t stand in those seats because if we do, the older ones insult you…and of course, the bus girl doesn’t go tell them anything because they are so powerful.”*

(Focus group 1)

### 3.4. Domain: The Victim

Within this domain, we identified two general core ideas related to the perception of victims’ weakness and the effects of bullying on well-being—which were prevalent among students. Additionally, ethnic victimization emerged as a typical core idea, while blaming the victim was categorized as a variant core idea.

#### 3.4.1. General

**Category: Victim’s weakness (or maybe not).** Variability emerges in the description of the victim. Some students believe that victims are fragile people because of several characteristics, such as physical or psychological. This reinforces the idea that victims are often identified by specific characteristics that distinguish them.

*“[…] maybe even those whose temperament is a little more sensitive than others”*

(Focus Group 1)

*“[…] against children who have physical difficulties …or …or …however they have difficulties in something”*

(Focus Group 6)

*“[…] those who are more…weaker…maybe who are not very fashionable right now, maybe they’re being made fun of because of the way they’re dressed, the mood they’re in…”*

(Focus Group 3)

However, others argue that the victim is not always weaker than the bully.

*“The victim is not always the weakest, i.e., he/she may even be stronger than the bully, only the bully thinks he/she is stronger”*

(Focus group 15)

*“Rather, the victim can have many friends, unlike the bully, who therefore may lash out at him precisely out of spite and a sense of injustice.”*

*“In my opinion, if the victim has friends and yet the bully does not, the victim is supported by these friends […] with him [the bully] there is no one… [but he still does it because] he wants justice”*

(Focus group 15)

**Category: The effects on victims’ well-being.** Students are aware of the profound effects bullying can have on victims’ well-being, including the risk of suicide.

*“[These experiences] are painful experiences […] traumatic […] that demoralize you […] maybe they don’t even make you leave the house anymore because you are afraid.”*

(Focus Group 9)

*“There are people who because of bullying have also had many problems of other kinds, like… there are people who have fallen into depression […] people who have also committed suicide after…”*

(Focus Group 3)

*“[…] however, within you remains the thing that a certain thing has been done on you”*

(Focus Group 12)

#### 3.4.2. Typical

**Category: Ethnic victimization.** Victims may be targeted specifically due to ethnic bullying, which is particularly noteworthy. As the number of people from diverse backgrounds increases, ethnic bullying has also become a significant issue in Italian schools (Basilici et al., 2022).

*“Victims of bullying are fragile people, people who appear weaker than others but actually are not, for example, those who belong to a different ethnic group…”*

(Focus Group 6)

*“[Bullies pick on] … for example those with a different skin color”*

(Focus Group 10)

*“My best friend, who is darker skinned, in elementary school, one of our classmates used to insult her because of her skin color.”*

(Focus Group 8)

#### 3.4.3. Variant

**Category: Blaming the victim.** In some focus groups, victim blaming emerged.

*“At the end, if one is bullied, it is his/her fault because he probably said something bad”*

(Focus Group 2)

*“If you can’t defend yourself anyway, it’s also your fault, i.e., at some point you have to defend yourself”*

(Focus Group 7)

### 3.5. Domain: The Other Roles

Students acknowledge additional roles that, while not directly engaged, play integral parts in bullying dynamics. However, in all focus groups, the characteristics of the bully and the victim were discussed extensively, while other roles were mentioned less frequently. As a result, we did not identify “general core ideas” for these additional roles. This suggests that bullying is perceived as a primarily dyadic interaction, involving the bully and the victim. This perception contrasts somewhat with our knowledge, which emphasizes bullying as a group phenomenon (Salmivalli et al., 1996) but is in line with previous literature (e.g., Guarini et al., 2019).

#### 3.5.1. Typical

**Category: Bystanders.** Those who witness a bullying incident can either actively intervene or passively observe, significantly impacting the situation’s dynamics. Some students recognize that bystanders have the power to stop the action through their intervention, while others blame them for their silence. They suggest that not intervening is to automatically become “pro-bully”.

*“[Also involved are] those who watch the bully…those who are around the event, the situation, I guess, the bully is beating the victim, and there are bystanders who help the victims…”*

(Focus Group 1)

*Let’s say, a person gets beaten up in the bathroom and there is a bystander […] he doesn’t say anything, and maybe he even makes a video, and the bystander is at fault, he has more or equal blame with those who are beating him… he’s complicit.*

(Focus Group 11)

*“He beat me up…however, I didn’t get hurt, I didn’t get hurt at all, however…it was also to say that anyway they were all keeping quiet…”*

(Focus Group 14)

It is also acknowledged that sometimes passive bystanders may behave that way out of fear.

*“If, for example, I saw a boy or girl being bullied, I wouldn’t go immediately to help him/her, because if more than two or three people were targeting him/her, they could beat you up…you would be afraid then…”*

(Focus Group 9)

**Category: Pro-bullies.** In some situations, bullying involves not only the bully, but also the pro-bullies who indirectly contribute, consciously or unconsciously, by supporting the bully through their actions or inactions. They may be a friend of the bully or other students involved in the bullying dynamic, supporting him/her for fear of retaliation against themselves.

*“Then there are other people who, in order not to get involved and not to become what is being bullied, join the bully”*

(Focus Group 3)

*“[Bullying] is a bad action, which usually involves many individuals that are: the bully, the bullied, and sometimes there are also helpers of the bully”*

(Focus Group 15)

#### 3.5.2. Variant

**Category: Defenders.** In some situations, there may be people who take a stand not only for the bully, but also for the victim. Although less mentioned, some students also refer to the role of those who join with the victim to comfort and help him/her cope.

*“There are the people who help… who try to get the person who is undergoing the things that the bully is doing through the moment […] so maybe they comfort him/her”*

(Focus Group 5)

*[…] however, his friends, since they knew that he would not arrive alone (the bully), joined with him, joined with my brother to go….” “To help him?” “Yes, in fact, when they saw them coming … there were five of the bully’s friends; instead, those with my brother were about ten and they ran away (the bully and his supporters)*

(Focus Group 11)

### 3.6. Domain: Victims’ Coping Strategies

This domain focuses on coping strategies available to victims. Five distinct core ideas have been identified: the first three are broadly applicable (“Ignore with Nonchalance”, “Distancing”, “Seeking Social Support”), while one is typical (“Victim’s Retaliation”) and another is a variant (“It Depends”).

#### 3.6.1. General

**Category: Ignore with nonchalance.** Victims may ignore the bully’s actions or provocations, attempting to diminish the bully’s satisfaction by eliciting a reaction. It is a method to disempower the bully by not providing the desired response. In this sense, it is important to highlight that this type of strategy is not avoidance, but rather it represents a deliberate nonchalance, already found to be mentioned as effective in other studies (Camodeca & Goossens, 2005; Widiharto et al., 2020).

*“In my opinion, the best way to beat a bully who insults you is to give a damn because the bully likes to see you angry or sad, so if you give a damn, the bully will stop after a while…”*

(Focus Group 6)

*“Taking the bully “by exhaustion”: not reacting to provocation so much as he gets tired after a while”*

(Focus Group 14)

**Category: Distancing.** In contrast, some students suggested employing avoidant strategies to manage bullying. While this coping strategy is also identified in the literature, it may not effectively address the bullying situation.

*“There is like if you see those people you’d rather hide so they don’t find you”*

(Focus Group 3)

*“The victim does not seek help from others because he or she is afraid of being threatened or making the situation even worse”*

(Focus Group 16)

**Category: Seeking social support.** In addition, victims often seek support from friends, family, or other networks for assistance or guidance, relying on external support to cope with the bullying experience. This type of coping strategies can help victims navigate the challenges of bullying, reduce stress levels, and promote better psychological adjustment (Garnefski & Kraaij, 2014).

*“I talk to someone, get advice… I tell an adult first”*

(Focus Group 6)

*“I ask for help to the parents, maybe they could call a psychologist”*

(Focus Group 3)

#### 3.6.2. Typical

**Category: Victim’s retaliation.** In other cases, victims may react to mistreatment with aggressive behaviour, using as much physical or verbal violence.

*“A friend of mine would sometimes even insult me and another and I would throw him out we would beat him up, but it was for his own good otherwise, he didn’t understand”*

(Focus Group 17)

*Um…for example, if any person here outside the school throws a punch at me, um…and it’s just him, but he just gives it to me on a whim, as if to say “Ah, I punch you because you deserved it” um…. and for example, I, I don’t know, I break my nose, then…I wouldn’t know how to respond because, in that case, I would be tempted to throw him a right hand that…that…that literally…because there just would really throw some strong things.*

(Focus Group 4)

#### 3.6.3. Variant

**Category: Talk with the bully.** In some cases, victims may directly address the bully to stop the mistreatment, assert boundaries, or seek resolution, taking a proactive approach to deal with the situation.

*If I respond to violence with more violence, the situation does not get fixed, because, in the end, we both end up in the middle: maybe it ends up with a suspension, or we end up in the hospital because we get hurt; in fact, the situation gets worse… I would try to talk to the person who is the bully and try to reason with that person.*

(Focus Group 13)

**Category: It depends.** It is interesting to note that victim coping strategies could vary in function of the type of bullying or the number of people present.

*“Mm… If one person insults me, I can confront him, you listen to him and then you don’t give any weight to what he says. The situation changes when eight people for example insult you; you have to intervene”*

(Focus Group 15)

### 3.7. Domain: Bystanders Coping Strategies

This domain addresses coping strategies that can be implemented by individuals who are not directly involved in bullying incidents. Like what happened about roles, students frequently talk about coping strategies that victims can use but struggle to identify strategies for bystanders. As a result, the core ideas we identified in this area are either typical or variant rather than general.

#### Typical

**Category: I will protect you.** Some bystanders opt for direct involvement, intervening physically or verbally to confront the bully or support the victim. They take an active stand against bullying behaviour.

*So… They got into a fight and so I went to the field and said “What are you doing” right? Then the high school girls came in and they said…they literally grabbed me and pulled me aside, and they said, “Stay here we want to watch the fight” and I stepped aside*

(Focus Group 9)

Other bystanders opt for a social intervention strategy, trying to talk to the victim and offering help, for example, suggesting that he or she turn to an adult to cope.

*“I wanted to say that anyway, for example, I would also talk to the victim, trying to convince them that maybe if you tell a person, who is an adult or teacher um…the problem could also be solved”*

(Focus Group 1)

**Category: I will protect myself.** Finally, bystanders might also hesitate to get involved due to fear of being drawn into the conflict or sustaining harm. This fear leads them to choose not to intervene, adopting a passive stance in the situation.

*“If I witnessed a fight, where one person beats up another, I’m not sure I would intervene”*

(Focus Group 7)

*“I wouldn’t get in the way for any reason because then I would end up in a fight myself”*

(Focus Group 11)

## 4. Discussion

This study employed consensus qualitative research to explore bullying. We identified seven key domains involving general, typical, or variant categories. The first domain focuses on the definition of bullying, traditionally based on three key criteria: power imbalance, intentionality, and repetition, all considered equally important. However, our study found that only power imbalance emerged as a general category, while intentionality was viewed as a typical and repetition was classified as a variant category, emerging as the least significant criterion. This result aligns with both quantitative (Menin et al., 2021; Skrzypiec et al., 2023) and qualitative (Strindberg et al., 2020) research, which have confirmed power imbalance as the central factor in defining bullying rather than intentionality or repetition. Our study highlights how, in practice, the three criteria do not carry equal weight despite their theoretical importance. In addition, it is the first study to consistently demonstrate this result using a qualitative approach across a large number of focus groups, showing the relevance of power imbalance in real-time discussions among groups of adolescents. The priority of power imbalance over intentionality and repetition highlights the complex dynamics of bullying, where social hierarchies and group dynamics play a crucial role (Hellström et al., 2021). This raises questions about the adequacy of traditional definitions, which may not fully capture the experiences of those involved in bullying situations. Finally, the low emphasis on repetition aligns with a broader scientific debate questioning the importance of repetition in defining bullying (see Hellström et al., 2021).

The second domain concerns the different kinds of bullying behaviours. Our findings reveal that students predominantly perceive bullying as a physical and verbal phenomenon, with relational bullying being relatively infrequent and only mentioned in a few cases. This perception aligns with both qualitative and quantitative studies, which indicate that students consider behaviors like hitting, threatening, and name-calling as more severe than actions such as spreading rumors and social exclusion (Bauman et al., 2020; Thornberg et al., 2018). It is plausible that this perception arises from the fact that verbal and physical bullying are more immediate and visible forms of aggression, making them easier to recognize. In contrast, relational bullying, despite being frequently reported, operates in a more subtle and covert manner (J. Wang et al., 2009), making it less noticeable to others. Consequently, indirect bullying might be perceived as less severe (Chen et al., 2015) and has potentially become so embedded in everyday life that it is no longer recognized as a form of bullying (Bauman et al., 2020)***,*** leading to a diminished awareness of its impact and harmfulness.

The third domain focuses on the bully’s perspective. Bullies are often understood through the motives behind their behavior, with two main themes emerging. The first, “The compensation perspective”, suggests that bullies act out due to their negative feelings stemming from personal problems or dysfunctional family environments. They use aggression as a way to protect themselves, aligning with early theories that view the aggressiveness of people as a response to their own perceived weaknesses (Gondolf, 1985; Staub, 1989). However, evidence linking the compensation perspective to bullying is limited (Cook et al., 2010), challenging the notion that bullying primarily stems from the bully’s insecurities or personal issues, but rather it represents a strategy to obtain status and social recognition (Caravita & Cillessen, 2012; Søndergaard & Hansen, 2018). At the same time, the compensation perspective can be related to the broader concept of bully-victims, youth who are both victims and perpetrators of aggression. Studies show that children exposed to high levels of aggression and victimization often respond with further aggressive behaviors (Leadbeater & Hoglund, 2009). This cyclical dynamic reflects attempts to externalize distress and regain control after experiences of abuse or neglect (Evans et al., 2018; Perren et al., 2012; Söderberg & Björkqvist, 2019), and evidence indicates that bully-victims face particularly negative long-term outcomes (Bettencourt et al., 2023). The second general category relates to moral disengagement, a key factor in becoming a bully or supporting bullying (Bjärehed et al., 2021). Moral disengagement allows individuals to detach from moral standards without experiencing remorse, guilt, or self-condemnation (Thornberg & Jungert, 2013). This enables behaviors such as justifying harmful actions as harmless fun or rationalizing them by blaming the victim, making it easier for individuals to participate in or support bullying. Although less spread, some students recognize that bullying is wrong since they fear retaliation rather than moral reasoning. This suggests that the deterrent against bullying is based more on the risk of consequences than on an understanding that bullying is inherently wrong, highlighting the need to cultivate a deeper sense of ethical responsibility. Finally, the perception of the bully’s dominance over adults is a less commonly mentioned but notable observation. Some students noted that bullies could instill fear even in authority figures, underscoring the broad impact of bullying on the school environment. This dynamic creates a climate of fear that affects not only individual victims but also peers and adults, reinforcing the bully’s power.

The next domain pertains to victims, where we identified two general categories the perception of victims’ weakness and the effects of bullying on well-being. In terms of perceived weakness, both quantitative and qualitative research indicate that victims are often perceived as physically weak or somehow different, which can contribute to their victimization (Celik et al., 2023; Strindberg et al., 2020). Interestingly, some students suggested that the victim’s perceived weakness might be deceptive, noting that victims might actually have more friends than the bully, potentially explaining the bully’s behavior and connecting with the need for belonging previously discussed. Regarding well-being, students recognize that bullying can have severe consequences, including leading to suicide. This awareness aligns with existing literature, which has long highlighted the profound effects that victimization can have on individuals (Savahl et al., 2019).

Ethnicity-based bullying is cited as a typical category within the victim domain. Notably, following the COVID-19 pandemic, there has been a rise in discriminatory behaviors targeting individuals from ethnic minority groups (Bhanot et al., 2021). Currently, skin color and nationality rank as the second most common reasons for bullying (UNESCO, 2019). Finally, victim blaming, identified in some focus groups, may be linked to moral disengagement that often stems from cognitive biases and social norms, where victims who do not conform to societal expectations are more likely to be blamed for their victimization. Reasonably, empathy and social norms reinforcing victim-blaming can be an effective deterrent.

The next domain focuses on the other roles, with the pro-bullies and the passive bystanders mentioned as typical, and the defender mentioned just in a few cases. This pattern reflects a broader tendency to view bullying primarily as a dyadic interaction between the bully and the victim, as already demonstrated through drawing analysis (Bosacki et al., 2006), questionnaires (Guarini et al., 2019), and innovative techniques like eye-tracking, which has shown that, on an implicit level, bullies and victims attract more attention than other roles (Menabò et al., 2023). The greater emphasis placed on the bully and the victim in the students’ words confirms this tendency also at the explicit level, as observed in focus groups. In addition, the frequent mention of the pro-bully and outsider roles, compared with the defender, suggests that students may see the social environment around bullying as largely passive or even complicit rather than actively supportive of victims. This may indicate that the defender is undervalued or underrepresented in school social dynamics, highlighting the need for more focused efforts to promote and support this role.

The next domain concerns the use of coping strategies by victims of bullying. The most common strategies were “ignoring the bully with nonchalance”, “distancing”, and “seeking social support”. Although the first two strategies may seem similar, students perceive them differently. Nonchalance does not involve emotional and cognitive distancing but rather an attempt to ignore the aggressor and minimize behaviors that might encourage or reinforce bullying (e.g., Andreou, 2001). This strategy can effectively reduce or even stop victimization, especially among boys (Salmivalli et al., 1996). In contrast, when avoidance is associated with emotional and cognitive distancing, it tends to reinforce negative emotional states and makes the victim feel more out of control or ineffective (Murray-Harvey et al., 2012; Slee et al., 2017). This distinction between nonchalance and distancing is crucial, as it suggests that the effectiveness of coping strategies may depend not just on the behavior itself but on the underlying emotional and cognitive processes. Nonchalance could be a practical approach in some cases, particularly for those who can manage to disengage from the bully without internalizing the aggression. However, more passive coping strategies, such as distancing, are not always functional, but they can become dangerous, sometimes exacerbating negative emotions (Thornberg et al., 2020). This highlights the need for nuanced support strategies that help victims maintain a sense of control and agency. The final general coping strategy identified is seeking social support, which represents a widely used adaptive coping strategy (Kendrick et al., 2012; Nixon et al., 2020). Seeking social support aligns with broader research on resilience, where strong social networks are often linked to better outcomes for those experiencing adversity (Andreou, 2001). Retaliation has also been identified as a typical cate. Previous research has revealed that retaliation is a common coping strategy (e.g., Van Dijk et al., 2017), even though it is generally not effective (Fox & Harrison, 2021). The persistence of retaliation as a coping strategy raises concerns, as it not only fails to resolve bullying but can also escalate the situation, leading to further harm.

Finally, two variant coping strategies were identified: talking to the bully and recognizing that there is no one-size-fits-all approach to dealing with bullying, as the situation and the type of bullying influence responses. Talking to the bully could reflect an attempt by victims to assert control over the situation where they otherwise feel powerless. Engaging with the bully might also be an effort to humanize themselves in the eyes of the aggressor, hoping to reduce hostility by fostering understanding. However, this approach can be risky if not supported by broader strategies and a safe, supportive environment, without which an initial peaceful confrontation can lead to a negative escalation of aggressive behavior.

The last domain strategy pertains to the bystander coping strategy, though it is noteworthy that bystander strategies are not widely mentioned. Indeed, we identified only a typical and a variant strategy, with no general bystander coping strategy emerging. This absence may be linked to the way students conceptualize bullying primarily as a dyadic phenomenon between bully and victim, which reduces their perceived involvement and responsibility to act. On one side, there is the perspective that bystanders should intervene when they witness bullying. This intervention can take various forms, such as directly confronting the bully, physically stepping in to protect the victim, or reporting the incident to trusted adults. However, this proactive behavior is not without risks, leading to the second contrasting strategy. Some students feel that intervening in defense of a victim could expose them to negative consequences, such as becoming targets of bullying themselves. As argued by other scholars (e.g., Pozzoli et al., 2012), this fear of retaliation can be particularly strong in environments where bullies hold significant social power or where there is little support from authorities. For these students, remaining passive is a self-protective measure, driven by the concern that standing up for others might result in their own victimization. This reasoning aligns with prior findings showing that adolescents evaluate the costs and benefits of intervening (Gini et al., 2008; Berkowitz, 2013). Moreover, there appears to be a discrepancy between intentions and actual behavior: while in hypothetical scenarios the majority of students report they would help the victim, in real-life situations a smaller proportion actually intervenes (Ma & Bellmore, 2016). In addition, the limited mention of bystander strategies suggests that many students may feel disempowered or unsure about how to respond to bullying effectively. Previous studies have underscored the need for interventions that not only encourage bystander intervention but also provide clear guidance and support for students who witness bullying so that they can effectively become upstanders (Hart Barnett et al., 2019; Vera et al., 2019). Empowering bystanders with the skills and confidence to act safely could significantly alter the social dynamics of bullying, reducing the power of bullies and increasing the protection for victims

### 4.1. Limitations

While this study provides valuable insights into students’ perceptions and experiences of bullying, important limitations must be acknowledged. First, the data were collected in classroom settings rather than using the traditional focus group approach. While this approach allowed for a broad range of perspectives, it may have limited the depth of discussion and the opportunity for students to share more personal or sensitive experiences. It is also possible that the need for group consensus within their class discouraged the expression of different views or led some students to shift the conversation to another topic. To address these issues, future studies could combine classroom-based discussions with smaller, traditional focus groups in order to balance breadth with depth. At the same time, adopting mixed methods, integrating qualitative approaches with self-report questionnaires or drawings, would allow for a broader and more nuanced understanding of students’ perspectives, particularly regarding bystander responses and bullying dynamics.

The second limitation concerns the breadth of topics addressed in this study. While our aim was to capture students’ spontaneous reflections, this approach may have limited the depth of exploration of certain themes that are particularly relevant, such as help-seeking behaviors and bias-based bullying. Future studies could therefore design focus groups or targeted qualitative inquiries specifically devoted to these areas, to better understand how students experience and interpret these issues and to generate more detailed and actionable insights.

Third, there are some limitations regarding the way coping strategies were elicited. Indeed, the lack of specific prompts may have constrained the depth of their reasoning. In addition, this question was asked at the end of the interview, after students had already discussed what bullying means to them and who is involved. This sequencing may also have influenced the content of their responses. Future research should consider designing more targeted prompts or placing questions about coping strategies earlier in the discussion.

In addition, our data were collected from a sample of Italian students, and it cannot be assumed that they are valid, for example, even for different age groups or nationalities. Future studies should replicate this work across diverse age ranges and cultural backgrounds.

Finally, while the study offers insights into the students’ perceptions of bullying, it does not capture the perspectives of other key stakeholders, such as teachers, parents, or school administrators. These perspectives could provide a more comprehensive understanding of the social dynamics and institutional factors that influence bullying behaviors and bystander interventions.

### 4.2. Implications

This qualitative research offers many reflections and implications. At the individual level, students emphasized power imbalance as the defining feature of bullying. Interventions should therefore prioritize reducing dominance and fostering balanced peer relationships. Social-Emotional Learning (SEL) programs can be particularly effective in this regard, as they cultivate empathy, respect, and mutual equality (Cipriano et al., 2023). Yet, focusing only on individual dynamics is not sufficient. Interventions must also address the broader social structures that sustain and reward aggressive behavior. Cultivating ethical responsibility is essential in this regard, as it helps counteract moral disengagement and victim blaming. A comprehensive approach should therefore combine efforts at multiple levels: targeting school norms, training bystanders, and fostering empathy. Evidence supports the value of such integrated approaches. Meta-analytic findings show that school-based programs can increase bystander intervention (Polanin et al., 2012). Programs such as STAC (Midgett et al., 2015, 2025), StandUp (Timmons-Mitchell et al., 2016), and NAB IT! (Nickerson et al., 2024) illustrate how coupling bystander skills with norm-based interventions can reframe bullying as a group phenomenon rather than a purely dyadic issue. Within this framework, empathy emerges as a unifying element. Students often described the harm to victims as their primary concern, indicating that fostering empathy toward those targeted could be a crucial component of anti-bullying efforts and a central focus of intervention programs. This is especially relevant in cases of ethnic-based bullying, where discriminatory behaviors may stem not only from individual biases but also from broader social dynamics.

Our findings also highlight that students most readily recognize overt forms of bullying, such as physical and verbal aggression, likely due to their immediate visibility and frequency. This underscores the critical need to continue addressing overt forms of bullying, like physical aggression, to prevent them from becoming normalized and accepted within school environments. However, focusing solely on these visible behaviors risks neglecting other equally harmful forms, such as relational bullying. Therefore, schools must not only focus on combating physical and verbal aggression but also work to raise awareness and educate students about the less obvious, indirect forms of bullying.

Concerning the roles, the underrepresentation of the defender raises concerns about the effectiveness of current interventions, indicating the need for more focused efforts to promote and support students in taking on this role. Indeed, fostering supportive peer environments and ensuring that victims feel they have allies they can turn to is crucial for preventing and countering bullying.

Finally, recognizing the variety of coping strategies available, interventions should provide a flexible range of approaches to help individuals effectively manage different situations (Kristensen & Smith, 2003). Retaliation as a coping mechanism should be discouraged, and more constructive strategies should be promoted for victims and bystanders instead. Indeed, providing bystanders with the skills to intervene safely may be an effective way to break the bully’s cycle of dominance and increase protection for victims.

## 5. Conclusions

To sum up, at a general level, the aspects that most clearly define bullying from students’ perspectives are the power imbalance and visible forms of aggression. General motives for bullying, such as moral disengagement and the desire to avoid their own problems, as well as the perception of victims as weak and the significant impact on their well-being, were strongly highlighted. It also became evident that victims may adopt various coping strategies, including seeking social support, distancing themselves, or simply ignoring the bullying. Additionally, we gained insight into less frequent but still typical factors, such as the perception of intentionality in bullying, concerns about potential retaliation from the bully, ethnic-based bullying, and the presence of “pro-bullies” and “bystanders” among the roles. The idea that victims might seek revenge and the possibility of bystanders intervening to defend the victim were also general considerations. Equally important are the variant aspects, which, although less evident, offer significant theoretical and practical insights. For example, repetition is rarely perceived as a defining characteristic of bullying, just as social exclusion is rarely recognized as a form of bullying. Moreover, the variant aspects help us understand dynamics that, although less common, can still serve as warning signs. Among these, the fear that the bully manages to instill even in adults and the belief that the victim ‘brought it on themselves’ are particularly concerning. Similarly, it is alarming that the role of the defender is mentioned far less often than that of bystanders and pro-bullies. In terms of coping strategies, victims rarely confront the bully directly, while few bystanders would choose to do nothing. Finally, it is interesting to observe that some students perceive the victims’ coping mechanisms as dependent on external influences, revealing a complexity of individual dynamics in their responses to bullying. To conclude, this study provides significant insight into students’ perceptions of bullying, shedding light on the complex nature of the phenomenon and the multifaceted dynamics that underpin it. By exploring both the general, typical, and variant aspects of bullying, this research deepens our understanding of how students interpret and consider bullying, offering a foundation for developing more targeted and effective interventions.

## Figures and Tables

**Table 1:** Domain and Core Ideas.

Domain	Category		
	*General*	*Typical*	*Variant*
Characteristics of bullying	“Dominance and Power imbalance”	“Intentionality”	“Repetition over time”
Bullying behaviours	“Physical harm” “Verbal aggression”		“Social exclusion”
The bully	“The compensation perspective” “Moral disengagement perspective”	“The repercussion perspective”	“The bully scares!”
The victim	“Victim’s weakness (or maybe not)” “The effects on victims’ well-being”	“Ethnic victimization”	“Blaming the victim”
The other roles		“Bystanders”	“Defenders”
		“Pro-bullies”	
Victims’ Coping Strategies	“Ignore with nonchalance”	“Victim’s retaliation”	“Talk with the bully”
	“Distancing”		“It depends”
	“Seeking social support”		
Bystanders’ coping strategies		“I will protect you”	“I will protect myself”

Note. This table presents the domains that emerged along with the categories identified for each domain. Each core idea is categorized as typical, general, or variant. No typical category was identified for the domain “Bullying behaviours”, and no general categories were identified for the domains “The other roles” and “Bystanders’ coping strategies”.

## Data Availability

The raw data supporting the conclusions of this article will be made available by the authors on request.
